# Vegetation response to precipitation anomalies under different climatic and biogeographical conditions in China

**DOI:** 10.1038/s41598-020-57910-1

**Published:** 2020-01-21

**Authors:** Zefeng Chen, Weiguang Wang, Jianyu Fu

**Affiliations:** 1 State Key Laboratory of Hydrology-Water Resources and Hydraulic Engineering, Hohai University, Nanjing, 210098 China; 2 College of Hydrology and Water Resources, Hohai University, Nanjing, 210098 China

**Keywords:** Ecosystem ecology, Environmental impact, Hydrology

## Abstract

Understanding precipitation-vegetation interaction is of great importance to implementing adaptation and mitigation measures for terrestrial ecosystems. Many studies have explored the spatial pattern of precipitation-vegetation correlation along the precipitation amount gradient. While the impacts of other precipitation characteristics remain poorly understood. Here, we provided a comprehensive investigation of spatiotemporal patterns of vegetation response to precipitation anomalies in China, using satellite-derived vegetation index and multi-source climate datasets for the years 1982–2015. Subsequently, we attempted to examine in detail what specific factors, climatic or biogeographic, are responsible for spatiotemporal patterns of precipitation-vegetation relationship. Results show that vegetation in Inner Mongolia Plateau is strongly affected by precipitation anomalies. Vegetation has a 1–2 month lag response to precipitation anomalies and is significantly correlated with 2–6 month cumulative precipitation anomalies. Seasonal differences of vegetation response are also remarkable. Moreover, the largest NDVI-precipitation correlation appears in areas with 150–500 mm of mean annual precipitation, 0.075–0.275 of fraction of precipitation days, and 19–23 of precipitation concentration index. More locally, the spatial distribution of NDVI-precipitation correlations is closely related to the vegetation type and elevation. The results can provide technical basis and beneficial reference to water resource and ecological management strategies in China for associated policymakers and stakeholders.

## Introduction

Sustained increase in Earth’s temperature driven by anthropogenic emissions of greenhouse gases is projected to alter patterns of global atmospheric circulation and hydrologic regimes^1,2^. For instance, both total cloud fraction and cloud top height have presented a robust increasing trend over wet areas worldwide^3^. The intensity of precipitation events and the frequency of extreme events have already increased across the globe and are expected to further increase^4,5^. These various kinds of climate fluctuations have triggered ongoing changes of terrestrial ecosystems, with implications for both the ecosystem structure^6^ and function^7,8^.

Vegetation, playing a significant role in the exchange of energy, water and carbon between the land surface and atmosphere, is the main component of the terrestrial ecosystem^9^. Substantial evidences suggest that the distribution, structure, composition, and diversity of vegetation populations and communities have being gravely impacted by global climate change^10–12^. Growing scientific and public concerns about the dramatic changes in vegetation cover resulting from climate change have thus incited a focus of researchers on the assessment of vegetation response to changes in air temperature^13,14^, precipitation^15,16^, solar radiation^17^, and sea surface temperature (SST)^18^. Among aforementioned climatic variables involving in vegetation-atmosphere interactions, precipitation variability is extraordinarily responsible for vegetation change due to the dominant role that precipitation plays in water availability which has been generally considered as the principal factor controlling ecosystem structure/dynamics and driving biological processes in over 40% of Earth’s vegetated surfaces^19,20^. Consequently, extensive studies have been specially conducted on precipitation-vegetation relationship in many areas of the world, such as Africa^21,22^, Central Asia^23,24^, and North America^25^.

In general, altered precipitation patterns have critical effects on vegetation communities, especially in terms of vegetation growth and development, although effects vary among different ecosystems and species^26^. For example, in a field experiment of typical Swiss grassland farming systems, below-normal precipitation has been proven to suppress vegetation growth, leading to a widespread grass withering^27^. Whereas, severely decreased precipitation tended to have no effect on vegetation growth and even conduce to the expansion of root system for increasing absorption of minerals and water, according to an experiment in the Ecological-Botanical Garden in Germany^28^. While a variety of eco-hydrological applications require data from broad spatial extents that cannot be collected by field-based methods^29^. With the development of satellite observation technology since the 20^th^ century, remotely sensed vegetation indices (VIs) from satellite data have been widely recognized as the effective approach to characterize vegetation dynamics at a larger spatiotemporal scale^30,31^. There has been a general consensus among scholars that vegetation response to precipitation variability exhibits tremendous spatial heterogeneity^32^. Considerable efforts have therefore focused on the interpretations for this phenomenon. For instance, White *et al*.^33^ showed that the dynamic response of terrestrial vegetation to precipitation perturbations over the United States depends on a variety of topographic attributes such as elevation, slope and aspect. Propastin *et al*.^23^ found that the great spatial variability of relationship between vegetation growth and precipitation in Central Asia can be attributed to distinct vegetation types. Chamaillé-Jammes and Fritz^34^ calculated the correlations between NDVI and precipitation fluctuations at the inter-annual time scale in eastern and southern African savannas, and discovered that mean annual precipitation (MAP) plays an active role in determining spatial distributions of vegetation sensitivity to altered precipitation regimes. Recently, in order to more comprehensively analyze the spatial pattern formation of vegetation response to precipitation variability, several studies tried to simultaneously consider the impacts of multiple external factors. Camberlin *et al*.^30^ examined the response of NDVI to precipitation variations in tropical Africa and detected that the spatially heterogeneous response of vegetation to precipitation is closely associated with MAP, vegetation type and soil properties. Hawinkel *et al*.^35^ analyzed the precipitation-vegetation relationship over East Africa based on an environmental effect model and demonstrated that vegetation sensitivity to precipitation variability is mainly controlled by MAP, vegetation type and elevation. Although aforementioned studies attempted to investigate the factors behind different vegetation responses to precipitation variability, except precipitation amount, the impacts of other precipitation characteristics (e.g. frequency, and distribution) on precipitation-vegetation relationship have never been concerned and further assessed.

China, a typical monsoon-controlled country, has the world’s third largest territorial area, with climate ranging from tropical to cold temperate and from rain forest to desert, as well as complex geographical conditions and a rich bio-diversity^36^. Along with a strong warming of China, the country has undergone significant alterations in precipitation patterns over the past few decades^37^. The long-term variations of precipitation, inflicting the increasing occurrences of precipitation extremes which have often been considered among the most severe natural hazards for China, are intimately related to the country’s economy and people’s life^38^. Investigation of vegetation response to precipitation variability across China will provide insight into the impact of precipitation fluctuations on terrestrial ecosystems and improve the regional management strategy to reduce ecological and economic losses. Hence, research on the precipitation-vegetation relationship in China has gradually received attention in the literature. For example, Li *et al*.^39^ analyzed the correlations between NDVI and precipitation during 1983–1992 and concluded that sensitivity and temporal response of vegetation to precipitation in China varies along the MAP gradient. Piao *et al*.^40^ also detected the critical effect of precipitation amount on precipitation-vegetation relationship in temperate grasslands in China and proved the existence of precipitation threshold of grassland biome. Wu *et al*.^41^ reported that the spatial pattern of sensitivity of vegetation response to precipitation in eastern China is partly driven by MAP. Gao *et al*.^42^ explored the climate change effects on vegetation activity in China and revealed that the spatial correlation between vegetation cover and precipitation variability may be altered due to varying climate features and vegetation types.

Despite recognizing the importance of precipitation characteristic to spatial pattern formation of vegetation response to precipitation, great attention in China has only been paid to the influence of precipitation amount on precipitation-vegetation relationship. To date, the effect of other precipitation characteristics such as precipitation frequency and precipitation distribution have not been included in previous studies and remain poorly understood. In practice, some scholars pointed out that changes in precipitation frequency and precipitation distribution can affect soil moisture variation, leaf carbon assimilation, soil carbon dioxide flux, vegetation physiology and morphology^11,26^. In other words, not only precipitation amount, discrepancies of precipitation frequency and precipitation distribution among different regions may also be the pivotal causes of spatially heterogeneous response of vegetation to precipitation. Therefore, in-depth studies on analyzing the spatial pattern of precipitation-vegetation relationship with considering the potential impacts of precipitation frequency and precipitation distribution are imperative. In addition, most studies that tended to evaluate the local effects (i.e. vegetation type, soil and topographic properties) on precipitation-vegetation relationship after avoiding the interference of precipitation conditions, only considered the influence of precipitation amount but ignored influences of precipitation frequency and precipitation distribution, increasing the uncertainty of the results. Hence, after completely eliminating the impacts of all three precipitation characteristics, re-examining the association between spatial distribution of vegetation-precipitation correlation and local factors is urgently needed.

To address aforementioned research gap, based on times-series of NDVI and climate datasets for the period of 1982–2015, this study aims (1) to characterize the spatial patterns of precipitation-vegetation relationship for identifying where and what extent vegetation growth is determined by precipitation in China, (2) to investigate the temporal patterns of vegetation response to precipitation anomalies with consideration of time-lag effect, varying precipitation cumulation periods and seasonal differences, and (3) to exhaust all possible causes behind the spatial distribution of correlations between vegetation and precipitation anomalies by taking more climatic (i.e. precipitation amount, frequency, and distribution) and biogeographical factors (i.e. vegetation type, topographic and soil properties) into account. The results provide a deeper understanding of vegetation response to current precipitation variability and vegetation-precipitation regulations, and help us to improve predictions of the future consequences of such variations in precipitation on ecosystems in China.

## Results

### Interannual variations in growing season NDVI and precipitation

**Figure 1:**
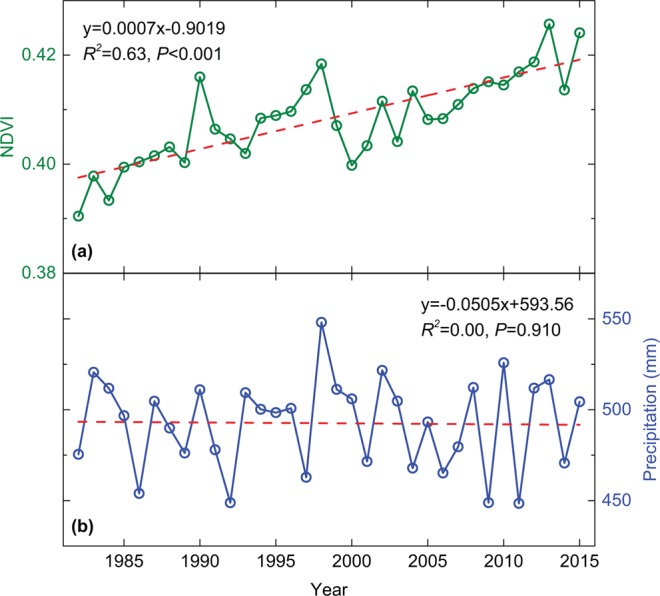
Interannual changes in area-weighted growing season mean normalized difference vegetation index (NDVI), and precipitation (mm) over the period 1982–2015 in China.

During the 34-year study period, mean growing season NDVI for China exhibited a significant increase (*R*^2^ = 0.63, *P* < 0.001) from 0.39 in 1982 to 0.42 in 2015, with a linear trend of 0.0007 per year (Fig. 1(a)). While growing season precipitation decreases slightly (*R*^2^ = *0.00*, *P* = *0.9*1*0*), with an annual decline of 0.0505 mm (Fig. 1(b)). Moreover, the pattern of precipitation fluctuation appears well in phase with NDVI. Growing season precipitation was relatively high in 1983, 1990, 1998, 2002, and 2013, coinciding with peaks of growing season NDVI. Similarly, the minima of growing season NDVI occurred in 1989, 2006, and 2014, corresponding with the low value of growing season precipitation over these years.

### Spatiotemporal patterns of precipitationvegetation relationship

**Figure 2:**
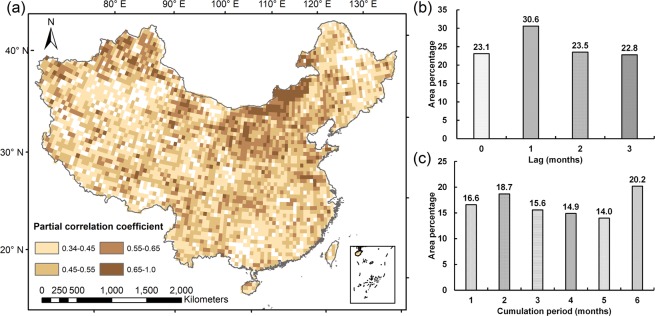
(**a**) Maximum partial correlation coefficient between precipitation and NDVI anomalies in China, areas without significant correlation (α = 0.05) are shown in white. (**b**) Percentage of temporal lag of the correlation analysis with best fit out of all partial correlation analyses with precipitation cumulation periods of 1 month in areas with significant correlation (α = 0.05). (**c**) Percentage of precipitation cumulation period of the correlation analysis with best fit out of all partial correlation analyses without time lags in areas with significant correlation (α = 0.05).

Figure 2(a) indicates the spatial map of maximum partial correlation coefficient passing the significant test between NDVI and precipitation anomalies, and determines locations where vegetation growth shows apparent response to precipitation variability. Areas without significant correlation (α = 0.05) for all 24 correlation analyses are shown in white. In all, 88.2% of the valid grids (grids with missing data or belonging to built-up areas were excluded, see Materials and Methods) display significant correlations. Highest correlations were found in northern China including almost the whole Inner Mongolian Plateau, Loess Plateau and the mountain ranges in the northwest. No significant correlations were found in the forest of the northeast, in some parts of the southern China as well as in some desert areas of the northwest.

To better understand the response of vegetation to precipitation anomalies, temporal lags and precipitation cumulation periods were assessed separately. Among all correlation analyses considering precipitation cumulation period of 1 month (first column in Table 1), the type of temporal lag that leads to the strongest correlation is shown in Fig. 2(b). The vegetation grids that have the greatest correlation with precipitation anomalies in the same month and do not show apparent lag effects, account for 23.1% of the total grids with significant correlations (α = 0.05). In fact, vegetation in most regions of China exhibits a certain lag effects related to precipitation anomalies, including 54.1% of the areas denoting time lag of 1–2 month. Among all correlation analyses without time lag (first row in Table 1), Fig. 2(c) reveals the length of precipitation cumulation period that is the most relevant for vegetation activity. The best fit length of precipitation cumulation period ranges between 2 and 6 months for most of grids with significant correlations. The proportion of grids where correlation between NDVI and 1 month accumulated precipitation anomalies delivers the strongest correlation is only 16.6%. That is to say, vegetation growth has a closer relationship with long-term cumulative precipitation anomalies.

**Table 1:** The design for analysis of temporal patterns (4 time lags × 6 cumulation periods).

Lag	Cumulation period					
	1	2	3	4	5	6
0	0	0–1	0–2	0–3	0–4	0–5
1	1	1–2	1–2	1–3	1–4	1–5
2	2	2–3	2–4	2–5	2–6	2–7
3	3	3–4	3–5	3–6	3–7	3–8

The numbers in the cells indicate the time interval for which precipitation is accumulated (0 indicates the current month, 1 indicates the first previous month, 0–1 indicates from current month to first previous month, etc.).

### Precipitation effects on precipitation-vegetation relationship

**Figure 3:**
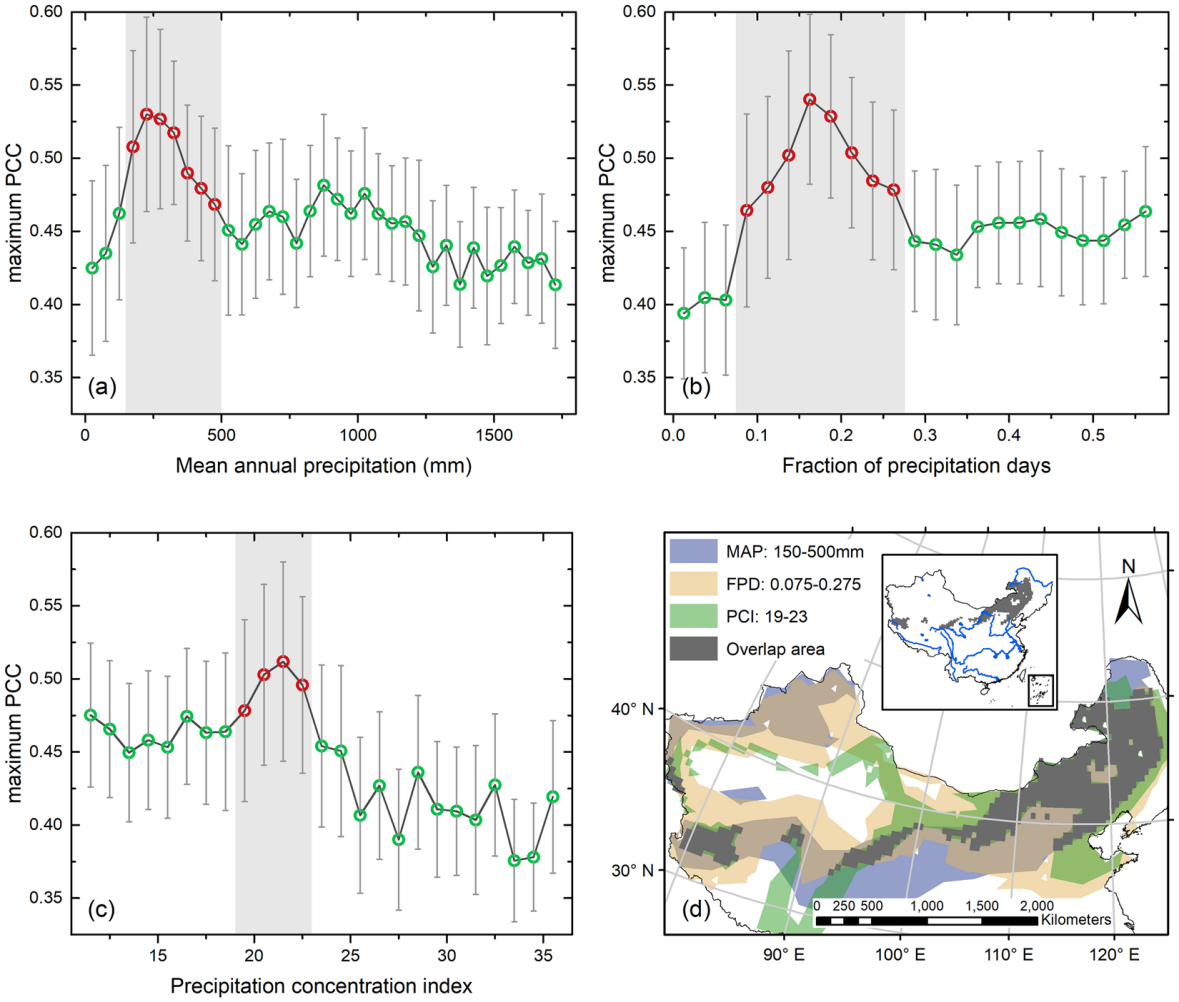
Relationship between binned averages of maximum partial correlation coefficient (PCC) and (**a**) mean annual precipitation (MAP), (**b**) fraction of precipitation days (FPD), and (c) precipitation concentration index (PCI) across China. Circles connected by a thick line are binned averages with every (a) 50 mm bins of MAP, (b) 0.025 bins of FPD, (**c**) and 1 bins of PCI, respectively. The PCC binned averages with statistical population less than 10 are excluded in the analyses. Error bars represent ± 1/2 SDs. (**d**) Spatial distribution of grid points that locate in the shadow range of (**a–c**) and their overlap area.

Three precipitation indexes, including mean annual precipitation (MAP), fraction of precipitation days (FPD), and precipitation concentration index (PCI) (see Materials and Methods), were analyzed with maximum partial correlation coefficient to investigate the potential effects of different precipitation characteristics on the vegetation response to precipitation anomalies at the national scale. By means of binned average analysis^43^, we found that the highest correlations between NDVI and precipitation anomalies occur in semi-arid areas with MAP around 150–500 mm, and the correlations reach a threshold at about 250 mm (Fig. 3(a)). When MAP is lower than 150 mm or higher than 500 mm, maximum partial correlation coefficient drops sharply and reaches mean values of 0.42 or less. Additionally, the results reveal significant effects of FPD and PCI. The statistical result has a significant correlation peak in areas with FPD around 0.075–0.275 (Fig. 3(b)). Meanwhile, a correlation peak can be seen ranging from PCI of 19 to 23 as shown in Fig. 3(c). For areas with higher PCI, mean values of maximum partial correlation coefficients decreased quickly until below 0.4. Therefore, grids with such precipitation conditions (150–500 mm MAP; 0.075–0.275 FPD and 19–23 PCI) have overall higher correlations between NDVI and precipitation anomalies than others. Based on aforementioned findings, the spatial distribution of areas where vegetation activity is largely impacted by precipitation anomalies can be depicted in Fig. 3(d).

### Local effects on precipitation-vegetation relationship

The presented analyses reveal that the strongest precipitation-vegetation relationship occur in China for areas with specific precipitation conditions (150–500 mm MAP; 0.075–0.275 FPD and 19–23 PCI). Nonetheless, pixel-based partial correlation coefficients highly scatter within these areas which were selected solely based on precipitation conditions. Precipitation condition may not be the only factor to influence precipitation-vegetation relationship. Therefore, local effects were added to explain the spatial heterogeneity of vegetation response to precipitation anomalies.

**Figure 4:**
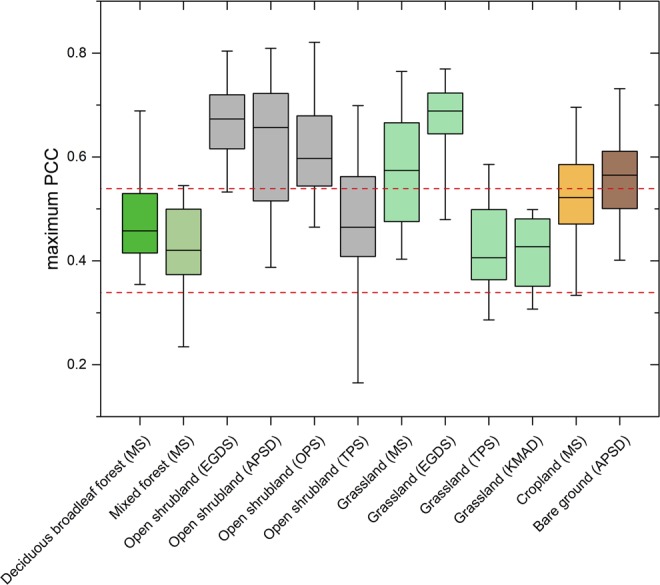
Box plots of maximum partial correlation coefficient (PCC) between precipitation and NDVI anomalies for different ecological zones (only areas with MAP: 150–500 mm, FPD: 0.075–0.275, and PCI: 19–23). Ecological zones which are represented by at least 10 grid points are shown in graph. Horizontal dashed lines (red) indicate 95% and 99.9% confidence line, respectively. Box plot elements: box = values of 25th and 75th percentiles; horizontal line = median; whiskers = values of 5th and 95th percentiles.

China has not only a large climate range, but also diverse vegetation types due to the large geographic size^44^. Precipitation-vegetation relationship across China may differ in the different vegetation types^25^. In this study, maximum partial correlation coefficient was stratified by ecological zones. Each ecological zone represents a unique group of vegetation type. Figure 4 shows the box-plot of maximum partial correlation coefficients between precipitation and NDVI anomalies for different ecological zones (only for areas in Fig. 3(d)). Furthermore, median value of maximum partial correlation coefficients for different ecological zones and precipitation conditions was tabulated in Table S1. Strongest correlations can be found for open shrubland, suggesting that such shrub-dominated vegetation is the most vulnerable to precipitation anomalies. When comparing different ecological zones of Mongolian steppe (MS), it is noticeable that tree-dominated ecological zones (i.e. deciduous broadleaf forest and mixed forest) show lower correlations than others (i.e. grassland and cropland) and median value of maximum partial correlation coefficients equal or below 0.45 (α = 0.01). As for vegetation in Tibetan Plateau steppe (i.e. open shrubland (TPS) and grassland (TPS)), it is less affected by precipitation anomalies than the same land cover/use class in other ecoregions. Besides, when comparing of median correlations among total grid points and two kinds of selected grid points (Table S1), we found that grid points selected by three precipitation indexes display the highest median correlations between NDVI and precipitation anomalies nearly in all ecological zones (except grassland (TPS)), further suggesting the necessity of considering FPD and PCI effect in vegetation response study.

**Figure 5:**
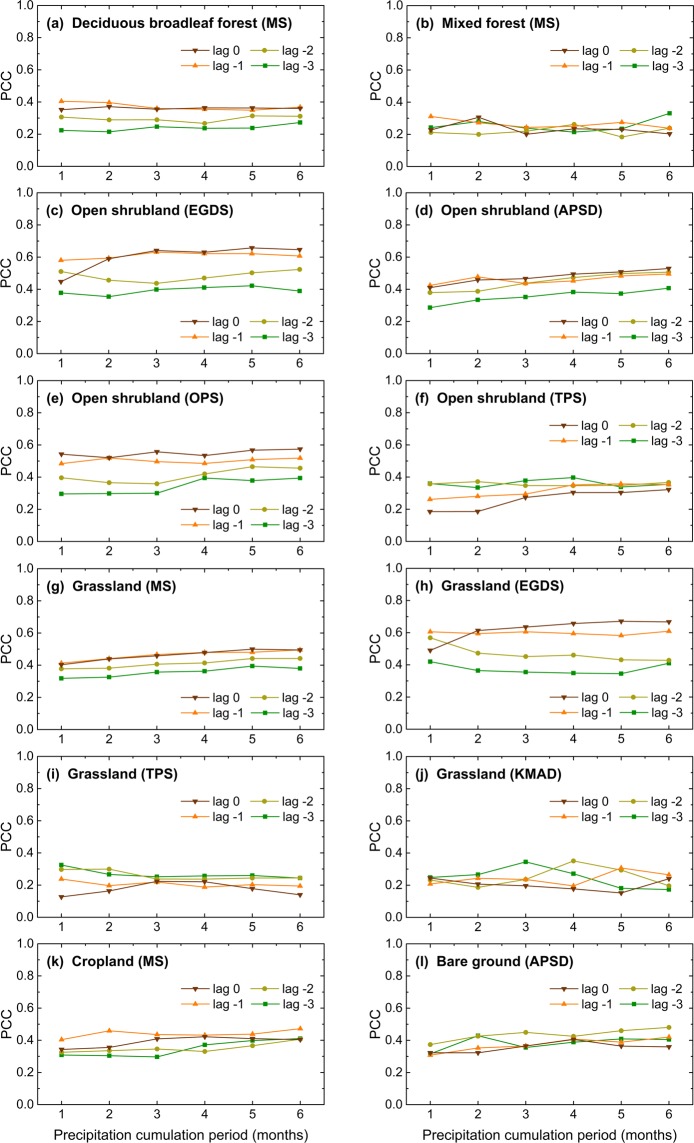
Temporal response patterns for different ecological zones (only areas with MAP: 150–500 mm, FPD: 0.075–0.275, and PCI: 19–23). The graphs show the median partial correlation coefficients (PCC) for each of the 24 correlation analyses. Colors represent the different time-lags (Lag 0–3 months).

Regarding the temporal response of vegetation to precipitation anomalies, it may be influenced by vegetation type as well. To validate this assumption, temporal patterns were further examined per ecological zone (Fig. 5). The median correlation coefficients for each of the 24 correlation analyses were used to illustrate the reaction of vegetation. Four ecological zones of Mongolian steppe (MS) are all characterized by a quick reaction with the strongest effect of the precipitation anomalies of the previous month (month -1) which denotes that the vegetation response to precipitation anomalies is delayed by 1 month. Nevertheless, for ecological zones of Tibetan Plateau steppe (TPS), the precipitation of the month -3 is the most important for vegetation growth, implying that the reaction of vegetation in these two ecological zones is slower than in others. Besides, in grassland (TPS), correlation with no time lag (lag 0) continuously increase until precipitation accumulates 3 months. However, in grassland (MS) and grassland (EGDS), longer accumulated (5–6 month) precipitation anomalies have the strongest impact on vegetation development. For cropland (MS), correlations stabilize or even decrease when precipitation cumulation periods become longer, suggesting that the effect of the length of precipitation cumulation period on vegetation is nearly negligible. Overall, it can be concluded from Figs. 4 and 5 that vegetation type is responsible for both spatial pattern of precipitation-vegetation correlation and temporal pattern of vegetation response to precipitation anomalies.

**Figure 6:**
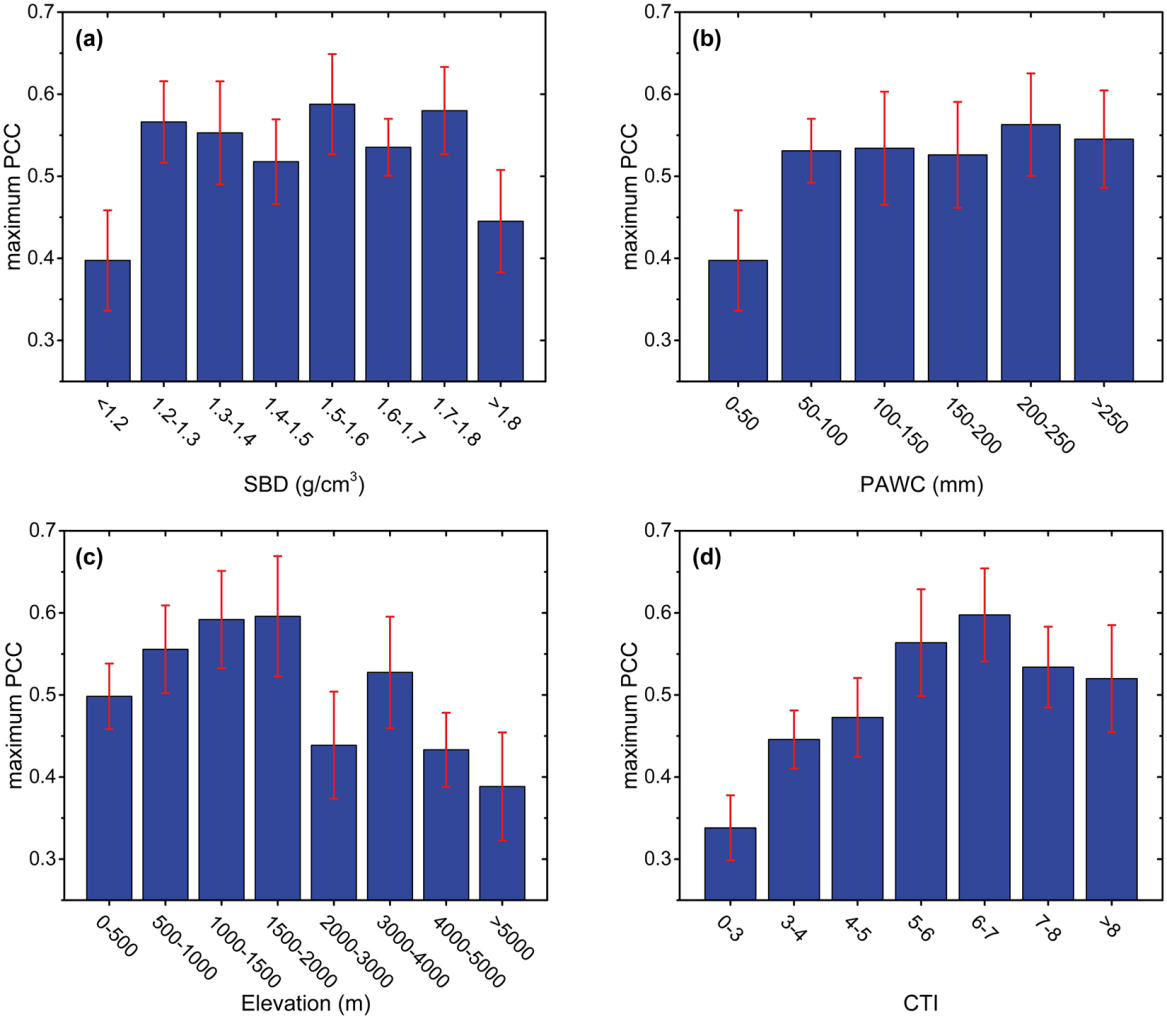
Relationship between maximum partial correlation coefficient (PCC) and (**a**) soil bulk density (SBD), (**b**) profile available water capacity (PAWC), (**c**) elevation, and (**d**) compound topographic index (CTI) for areas with MAP: 150–500 mm, FPD: 0.075–0.275, and PCI: 19–23. Boxes indicate the mean values, and error bars represent ± 1/2 SDs.

Soil and topography were also used to detect whether these local environmental factors contributed to vegetation response pattern. We examined the way that precipitation-vegetation relationship is modulated by four geographical factors at the pixel scale (Fig. 6). Soil-based factors such as soil bulk density (SBD) and profile available water capacity (PAWC) have little impact on correlation between NDVI and precipitation anomalies. PAWC classes exceeding 50 mm show quite similar mean values of maximum partial correlation coefficients. Elevation displays a more robust incidence on precipitation-vegetation relationship. The correlation peak for elevation ranges from 1000–2000 m. When elevation exceeds 2000 m, correlations drop sharply. As for the occurrence of relatively high correlation in elevation of 3000–4000 m, it may be closely related to the endemic species in Qaidam Basin, since grids with 3000–4000 m altitude are mainly located in Qaidam Basin semi-desert (QBSD). Additionally, it can be observed that mean value increases (from 0.42 to 0.48) along with the compound topographic index (CTI), and reaches peak for CTI ranging from 6–7.

### Seasonal differences of precipitation-vegetation relationship

**Figure 7:**
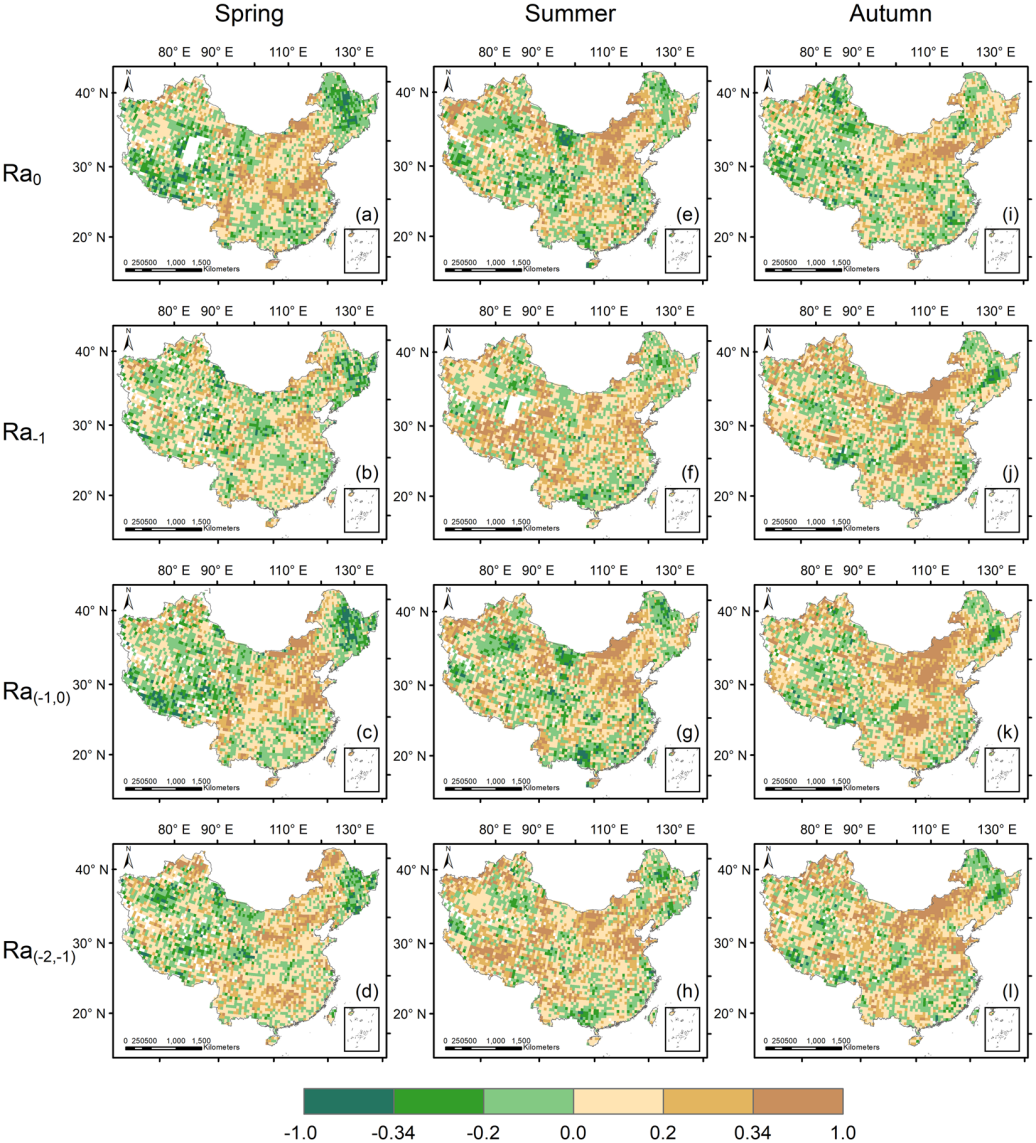
Spatial patterns of partial correlation between seasonal precipitation and NDVI anomalies in China (Ra_0_: partial correlation coefficient between seasonal NDVI and precipitation anomalies in the same season; Ra_-1_: partial correlation coefficient between seasonal NDVI and precipitation anomalies in the previous season; Ra_(−1,0)_: partial correlation coefficient between seasonal NDVI and precipitation anomalies from the previous season to the current season; Ra_(−2,−1)_: partial correlation coefficient between seasonal NDVI and precipitation anomalies in the previous two seasons).

In order to investigate the seasonality of precipitation-vegetation relationship, we further analyzed the seasonal NDVI instead of the growing season NDVI. Figure 7 illustrates spatial distributions of correlation coefficients between seasonal NDVI and precipitation anomalies at the national scale. Comparing the results of Ra_0_ in Fig. 7, the highest correlations between seasonal NDVI and the current season’s precipitation anomalies were observed separately in Hengduan Mountains of the southwest (25–32°N, 97–102°E), in the central part of Inner Mongolian Plateau (40–45°N, 105–115°E) and in Yanshan Mountains (40°N, 115–120°E). In spring, significant positive correlations occur in lower latitude areas than in summer. For example, in Huai River Basin (31–36°N, 112–120°E), there is a significant positive correlation between spring NDVI anomalies and concurrent precipitation anomalies (plot a), but a negative correlation appears in summer (plot e). As for Kashgar Oasis (40°N, 75°E), only vegetation growth in summer is controlled by current season’s precipitation (plot e). Comparing with other two seasons, high-related areas in autumn have a smaller range, which concentrates in northern part of North China Plain and surrounding mountains (38–40°N, 110–120°E) (plot i). In addition, spatial patterns of correlation coefficients between seasonal NDVI and precipitation anomalies in the preceding season were plotted in Ra_−1_ in Fig. 7. Spring NDVI anomalies are highly correlated with the previous season’s precipitation in North China Plain (35–40°N, 115–120°E) while it has less significant correlation with non-growing season precipitation than with spring precipitation in Inner Mongolian Plateau (40–50°N, 106–120°E) (plots a and b). A strong linkage between summer NDVI and spring precipitation anomalies occurs in some high-altitude regions in the northwest, including Qilian Mountains (36–39°N, 95–100°E), Tibetan Plateau (28–35°N, 85–100°E) and Tianshan Mountains (42–43°N, 80–85°E) (plot f). In autumn, especially for Helan Mountains (39°N, 106°E) and upper Yangtze River Basin (28–32°N, 105–112°E), vegetation growth is largely determined by summer precipitation variability (plot j), while in the same area, a weak correlation was obtained between spring NDVI and winter precipitation anomalies (plot b), or summer NDVI and spring precipitation anomalies (plot f).

Considering the impact of precipitation cumulation period, correlation analyses between seasonal NDVI and precipitation anomalies accumulating two seasons were also employed (Ra_(−1,0)_ and Ra_(−2,−1)_ in Fig. 7). Although the positive correlation between seasonal NDVI and precipitation anomalies from the previous season to current season in Inner Mongolian Plateau is always notable whatever the season, the range of high-related areas becomes wider as time goes from spring to autumn (plots c, g and k). Furthermore, significant negative correlations between spring NDVI and precipitation anomalies from winter to spring were found exclusively in Tibetan Plateau and Northeast China (plot c). Nonetheless, negative correlations between summer NDVI and precipitation anomalies from spring to summer scatter in many parts of the country, including Guangxi Province in the southwest (22–26°N, 105–110°E), northern margin of Hengduan Mountains (30–32°N, 97–102°E), Badain-Jaran Desert (40–42°N, 98–104°E) and so on (plot g). Due to the positive effect of summer precipitation anomalies on autumn vegetation activity in upper Yangtze River Basin (plot j), a similar strong correlation between autumn NDVI and accumulated precipitation anomalies from summer to autumn was identified in this basin (plot k), where weak correlation was found both in spring and summer (plots c and g). Only by considering time-lag and the effect of precipitation cumulation period at the same time, we could find the occurrence of significant positive correlations in Daxing’an Mountains in the northeast (45–52°N, 118–123°E) (plot d). Additionally, autumn NDVI anomalies of upper and middle Yangtze River Basin (28–32°N, 105–116°E) exhibit highly positive correlations with precipitation anomalies accumulating from spring to summer (plot l).

Variations in the seasonal precipitation-NDVI correlations may mainly result from differences in regional climates and vegetation structure. Table S2 summarizes median partial correlation coefficients between seasonal NDVI and precipitation anomalies for different precipitation conditions and ecological zones. Comparing with the result obtained at the national scale (first row in Table S2), semi-arid areas with MAP ranging from 150 to 500 mm or with stricter precipitation conditions (MAP: 150–500 mm; FPD: 0.075–0.275; PCI: 19–23) exhibit overall stronger correlations between seasonal NDVI and precipitation anomalies (second and third rows in Table S2) no matter what the season and time-scale. Under certain precipitation conditions, seasonal pattern is changed. For example, summer NDVI anomalies under specific precipitation conditions show higher correlations with precipitation anomalies in summer than in spring. However, at the national scale, vegetation growth in summer has a closer relationship with precipitation anomalies in spring. Even though under the same precipitation conditions, relationship between seasonal NDVI and precipitation anomalies is determined by vegetation type. For all selected ecological zones, spring NDVI is correlated only weakly with non-growing season precipitation, and autumn NDVI is little affected by autumn precipitation. Tree-dominated ecological zones show non-significant correlation no matter what season, and even exhibit negative correlations between spring NDVI and concurrent precipitation anomalies. Additionally, vegetation growth of open shrubland is the most vulnerable among all vegetation types. For open shrubland, vegetation growth in autumn is largely controlled by summer precipitation. For grassland (MS) and grassland (EGDS), summer and even spring precipitation anomalies have significant influence on autumn vegetation activity. While for high-elevation grassland ecological zones (i.e. grassland (TPS) and grassland (KMAD)), vegetation has tiny correlation with precipitation anomalies. Similar pattern can be found in cropland (MS), despite the completely different internal mechanism.

## Discussion

### Spatiotemporal patterns of vegetation response to precipitation anomalies

The correlation analyses provided an overview of characteristics of precipitation-vegetation relationship in China. Significance and value of the maximum partial correlation coefficient reflect where and how strongly vegetation activity is influenced by precipitation anomalies. As shown in Fig. 2(a), most regions of China are dominated by significantly positive correlations between NDVI and precipitation anomalies. Vegetation growth in such regions with the high maximum partial correlation coefficient is greatly influenced by precipitation anomalies. In general, it is believed that the significant positive correlations between growing season NDVI and precipitation occur mainly in arid and semi-arid areas where water supply is limited, such as northern China^42^, Central Asia^24,45^ and African Sahel^46^. With the increase in precipitation variability, these high-related areas are ecologically fragile and prone to droughts due to their dependence to precipitation. No obvious correlation was observed in some humid areas, especially in southeastern China, suggesting that water availability is no more a constraint in these humid areas and adequate soil moisture could reduce the dependence of vegetation on precipitation^36,47^. Furthermore, weak correlations between NDVI and precipitation anomalies also appear around the desert in the northwest, and primary forest of Xiaoxing’an Mountains in the northeast. Therefore, it is reasonable to consider that vegetation response to precipitation anomalies can be influenced by some other factors. The impacts of climatic and biogeographical factors would be further discussed.

Moreover, the results indicate that the delay between NDVI and precipitation anomalies is pronounced in large parts of China. Temporal response patterns show a temporal lag between precipitation anomalies and vegetation activity of 1–2 months. Reaction of vegetation is strongest for precipitation anomalies accumulated over periods of 2 and 6 months. 1-month lag shows a high correlation between NDVI and precipitation anomalies, which explains the reasonability of the high percentage for the 2-month precipitation cumulative period. With respect to the high percentage for 5 and 6 months cumulative period, we can find the reasons based on the results at the seasonal scale. According to Fig. 7, comparing with the results of Ra_(−1,0)_ and Ra_0_, we discovered that long-time cumulative precipitation anomalies ranging from 5–6 months have more stronger effect on vegetation growth than short-time (2–3 months) in some parts of China, especially in Loess Plateau, Yellow River Delta, and upper Yangtze River Basin, explaining result of Fig. 2(c) that 34.2% of grid points deliver the strongest correlation between NDVI and 5–6 month accumulated precipitation anomalies. The hysteresis has been broadly reported all around the world, implying the role of storage in both the soil and ecosystem, in modulating vegetation response to precipitation variability^22,32^. For instance, lags of 1–2 months were identified in studies of southeastern North America^18^. Wang *et al*.^48^ discovered time lags of 4 weeks and precipitation cumulation periods of 4 or 5 biweekly periods in the central Great Plains, USA. Souza *et al*.^49^ found that lags range from 0 to 2 months in Northeast Brazil. Also, plenty of analyses have been performed in China, although most of them focused on smaller regions. Zhong *et al*.^50^ noted lags of 1–2 months for Tibetan Plateau. Jiang *et al*.^47^ identified time-lag of 1 month for the eastern and northeastern of China, supporting our findings that vegetation growth is primarily determined by the precipitation within the previous month in China, as a whole.

As a consequence of the vegetation phenology, the effect of altered precipitation on vegetation can be significantly different according to growth phase^51^. In general, the vegetation growth in spring is weakly correlated with winter precipitation^52^. As for North China Plain, a high correlation between spring NDVI and pre-season precipitation anomalies was observed in Fig. 7(b), which is quite different from other regions. Due to the geographical location of North China Plain, winter precipitation falls as snow and further forms suitable snowpack which can increase temperature in shallow soil layers on account of the insulating effect of the snow, and therefore protect vegetation from chilling and freezing injury^28^. Spring temperature rise greatly motivates vegetation greening in spring rather than spring precipitation in large areas of China^53^, due that the warmer environment is critical for the germination of most of the vegetation types, while more precipitation can cooler surface or even cause flood, and thus restrain vegetation growth^40^. In addition, other studies revealed that spring precipitation could largely affect soil moisture and further affect vegetation growth in summer for alpine ecosystems^54^, consistent with our results (Fig. 7(f)). On account of the influence of Asia monsoon, most of precipitation falls during the summer^50^, and water availability is mostly determined by summer precipitation in China. As documented by Ren *et al*.^55^ in the northwestern China, our results implied that summer precipitation anomalies strongly correlate not only with NDVI anomalies of summer, but also with that of autumn. In other words, pre-season precipitation can alleviate water stress on vegetation growth during autumn, decrease the risk of chlorophyll degradation, and slow down the timing of leaf senescence^56^. However, autumn precipitation anomalies have little effect on corresponding NDVI anomalies, in agreement with previous studies^48,54^.

### Influences of climatic and biogeographical factors

Various types of climate, diverse vegetation coverage, and complicated topographical features in China, may lead to the uncertainty on the prospect of ecosystems under changing precipitation regimes^36^. In this study, the results agree on the significant contributions of precipitation characteristics, vegetation type and topography to great spatial heterogeneity of precipitation-vegetation relationship across the country.

It is widely stated that the spatial patterns of precipitation-vegetation relationship closely reflect the MAP^57^. In our study, results show that the maximum correlations between NDVI and precipitation anomalies occur in areas with MAP between 150 and 500 mm (Fig. 3(a)). Beyond 500 mm of MAP, correlation decreases gradually, indicating that sufficient average precipitation can be obtained to induce regular leafing therefore photosynthetic activity behaves insensitively to precipitation anomalies^57^. In highly arid areas below 150 mm of MAP, low correlations were also observed which may be associated with low or even absent vegetation coverage and short-time vegetation “green-up”^45^. The remotely sensed signal is dominated by other surface properties, and can be strongly biased by atmospheric effects^30^. Additionally, in these arid areas, NDVI anomalies, showing up as slight variations in biomass, may also result in low correlations. Besides, Camberlin *et al*.^30^ demonstrated that the upper and lower bounds of significant correlations depend on the region. For East Africa, Hawinkel *et al*.^35^ discovered the peak in vegetation sensitivity is around 500 mm of annual precipitation. For Africa savannas, Chamaillé-Jammes and Fritz^34^ detected an inflection of the precipitation-NDVI relationship around 600 mm of MAP. Therefore, we pointed out that on average the precipitation-vegetation relationship becomes insignificant at the same threshold in China than in some zones of Africa. Simultaneously, we detected that the correlation between vegetation growth and precipitation anomalies in China also varies along FPD and PCI gradients, and proved the existence of FPD and PCI thresholds. Higher precipitation frequency (FPD > 0.275) standing for more rainy days has weak impact on the relationship between NDVI and precipitation anomalies. Furthermore, areas with lower FPD (FPD < 0.075) and areas with higher PCI (PCI > 23) were highly overlapped. As for PCI, high precipitation distribution results from extend periods of drought or intense precipitation events or a combination of both^58^. For areas with more uniform rainfall regime (PCI < 19), a relatively low vegetation response to precipitation anomalies suggested that vegetation with relatively stable water availability all year round can be buffered from the effect of precipitation anomalies. Areas with more concentrated rainfall (PCI > 23) corresponding to harsh environments, are mainly located in deserts (e.g. Taklamakan Desert, Kumtag Desert and Badain-Jaran Desert) and covered by desert vegetation such as desert grass and desert shrub. Thus, decreasing correlations in these deserts can be explained that (1) for desert grass, the response of vegetation to anomalous precipitation may be constrained by low meristem density and photosynthetic capacity^59^; and (2) for desert shrub, the direct effects of above-normal or below-normal precipitation on the growth of vegetation can be very weak owing to the dependence on groundwater^60^.

In fact, same as previous studies taking account of mean annual precipitation amount only^22,45^, our study aims to explore the association between the spatial distribution of NDVI-precipitation correlations and precipitation condition, and further determine what kind of precipitation condition that the largest correlation between vegetation growth and interannual precipitation variations is dominantly related to. According to the results in Tables S1 and S2, it is noticeable that areas with a given precipitation amount, frequency and distribution (i.e. 150–500 mm MAP; 0.075–0.275 FPD and 19–23 PCI) show overall higher correlations between NDVI and precipitation anomalies than areas only with a certain precipitation amount (i.e. 150–500 mm MAP), in all cases. In other words, benefit from the simultaneous consideration of the impacts of MAP, FPD and PCI, we found a more accurate precipitation condition where the highest precipitation-vegetation correlation is found. Besides, by means of such a more accurate precipitation condition, we could effectively reduce the interference of precipitation effects in our further analysis of the influences of local factors on precipitation-vegetation relationship.

Furthermore, the results obtained in this study demonstrated that vegetation type also is an important factor accounting for the spatial distribution of precipitation-vegetation relationship. For tree-dominated ecological zones, NDVI anomalies exhibit tiny correlations with precipitation anomalies (Fig. 4), even though the tree-dominated areas exclusively fall into the semi-arid regions, because trees with well-developed root systems are able to reach deep soil water resources and are weakly influenced by soil moisture variations. Similar observations were made by Li *et al*.^39^ in China, by Wang *et al*.^48^ in USA and by Camberlin *et al*.^30^ in Africa. By contrast, shrubland and grassland have much shallower root systems so that vegetation growth is highly affected by precipitation anomalies^39^. In cropland, a weak relationship between seasonal NDVI and precipitation anomalies (Table S2) can be attributed to human activity because water availability in cropland is sufficient and is uncoupled from *in-situ* precipitation with the help of water management. In fact, crop development mostly depends on agricultural irrigation as well as precipitation and snowmelt in upstream regions^45,61^.

Our results show that the vegetation response to precipitation can be modulated by local topography, which is in accordance with previous studies^31,33^. Interestingly, a correlation peak can be identified for elevation ranging from 1000 to 2000m (Fig. 6). In higher altitude areas, as the elevation increases, greater amounts of snow arise from increasing precipitation^62^. Ropars *et al*.^63^ deduced that deep snow cover leads to mechanical damage to stems and leaf buds, strongly limiting vegetation growth. Nevertheless, in lower altitude areas, more frequent anthropogenic activity disturbances^64,65^ and more water resources (e.g. fault springs)^66^ also weaken the effect of precipitation anomalies on vegetation. Besides, there is no doubt that soil properties such as SBD and PAWC would influence water storage and infiltration. However, considering limited runoff, vegetation in such water-limited regions is able to utilize almost all available precipitation regardless of soil properties^67,68^. Thus, differences in soil properties have weak impact on the maximum partial correlation coefficient between NDVI and precipitation anomalies.

## Conclusions

In this study, spatiotemporal patterns of vegetation response to precipitation anomalies were explored at the national scale, based on time series of NDVI and precipitation datasets during the period of 1982–2015. Meanwhile, the impacts of precipitation characteristics (i.e. precipitation amount, frequency, and distribution) on precipitation-vegetation relationship were evaluated. Finally, association between the spatial distribution of NDVI-precipitation correlations and local factors were examined in depth. Some interesting findings are obtained from this investigation as follows:

1. Vegetation in northern China, especially in arid and semi-arid regions, exhibits high correlation with precipitation anomalies. These areas are ecologically fragile and highly depend on precipitation, and thus ecological services such as forage supply and crop production are greatly affected by precipitation anomalies. Ecosystems in northern China should be paid more attention because of the increase in precipitation variability in the future.
2. 1–2 month hysteresis broadly exists in the response of vegetation to precipitation anomalies for 54.1% of the vegetated areas in China. Furthermore, 2–6 month cumulative precipitation anomalies has more significant impact on vegetation growth and development over large parts (83.4%) of China, than instantaneous ones. Seasonal differences of vegetation response are also remarkable. For example, summer precipitation anomalies are strongly correlated with NDVI anomalies of summer, but autumn precipitation anomalies have no obvious correlations with that of autumn. These results provide a basis for predicting vegetation anomalies by analyzing precipitation data.
3. Attribution analyses of spatial pattern of vegetation response to precipitation anomalies reveal that precipitation characteristics (i.e. precipitation amount, frequency and distribution), vegetation type and topography play a significant role in the great spatial heterogeneity of correlation between NDVI and precipitation anomalies across China. Specifically, we concentrated on the influences of precipitation frequency and precipitation distribution on precipitation-vegetation relationship, which has not been explicitly indicated in the previous studies. In addition, we identified the existence of precipitation thresholds in China. As expressed by precipitation indexes, the particularly strong correlation occurs in areas with MAP of 150–500 mm, FPD of 0.075–0.275 and PCI of 19–23. As for topography, the correlation peak for elevation ranges from 1000 to 2000 m. Thus, it is essential to properly identify ecological zone and meaningful influence factors when describing the complex spatiotemporal vegetation response for the assessment of climate change sensitivity.

## Materials and Methods

### Vegetation index

The NDVI has been widely used as a surrogate of vegetation dynamics^69^. The Global Inventory Monitoring and Modeling Systems (GIMMS3g.v1) NDVI dataset, derived from the Advanced Very High Resolution Radiometer (AVHRR) instruments on board National Oceanic and Atmospheric Administration (NOAA) satellites, was used for the analysis^70^. We selected the newly updated GIMMS3g dataset since it covered a longer period (1982–2015) and provided more complete NDVI record than other existing datasets^32^. Meanwhile, GIMMS3g was famous for high quality because it has been calibrated to eliminate noise produced by atmospheric effects, cloud cover, volcanic aerosols, effects of satellite drift, sensor view angles and so on^71^. Despite the efforts made in the data processing, there remain some biases in the dataset. Here, the original data (15-day interval at a 8-km spatial resolution) were resampled to 0.5° latitude and longitude resolution to suit the climate data, and were aggregated to a monthly temporal scale using the maximum value composite (MVC) technique which can largely remove atmospheric noise^72^. Next, we found the unexpectedly low values in some areas around water bodies and mountains, after the close examination of monthly NDVI dataset. Any grid point with one such abnormal value was set to missing, since it was recognized as possibly contaminated. In order to avoid the influence of winter snow and better reflect the growth status of vegetation^73^, our study only focused on NDVI during growing season (April-October). The spatial pattern of average NDVI in growing season is shown in Fig. S1(a). For further analysis, the growing season was divided into spring (April-May), summer (June-August), autumn (September-October). Seasonal NDVI values were generated separately through calculating averages of respective months. Anomalies were computed by subtracting 34-year mean monthly (seasonal) NDVI values from the current monthly (seasonal) values.

### Climate data

The monthly precipitation (PRE), temperature (TMP), and rainday-counts (WET) datasets used in this study were acquired from the Climatic Research Unit (CRU), version TS4.01^74^, covering our study period (1982–2015) with a 0.5° spatial resolution. The monthly 0.5° gridded solar radiation dataset was derived from a reanalysis product (ERA-Interim) produced by the European Centre for Medium-Range Weather Forecasts (ECMWF)^75^, which spanned from 1979 to present. Climate data for 1981 were also used since we took account of time-lag effect and precipitation cumulation period. To evaluate the reliability of gridded climate datasets, we analyzed the correlations between field observations and the corresponding gridded values. Observation dataset, collected from 614 well-distributed meteorological stations of the China Meteorological Administration (CMA), included daily records for precipitation, temperature, and sunshine hours (Fig. S2). Due to the lack of observed solar radiation data, we estimated solar radiation from sunshine hours by means of Angstrom formula^76^:

1 $${R}_{s}=({a}_{s}+{b}_{s}\frac{n}{N}){R}_{a}$$

where *R*_*s*_ is the solar radiation (MJ *m*^−2^ *d*^−1^), *n* is the actual sunshine hours (h), *N* is the maximum possible sunshine hours (h), *R*_*a*_ is the extraterrestrial radiation (MJ *m*^−2^ *d*^−1^), *a*_*s*_ and *b*_*s*_ are the Angstrom coefficients.

Average Pearson’s correlations of 0.89, 0.99, and 0.93 for precipitation, temperature, and solar radiation confirm the suitability of aforementioned gridded climate datasets for exploring climate-vegetation relationships at the regional scale. Considering temporal lags between climate and vegetation response, we separately calculated six anomaly datasets of precipitation, temperature, and solar radiation in monthly intervals to indicate deviations from the respective means. The anomaly composites were computed at each monthly time step as the climate anomaly data was considered to have six different periods defined as current month plus 0 to 5 previous months.

Three precipitation indexes including mean annual precipitation (MAP), fraction of precipitation days (FPD) and precipitation concentration index (PCI) were calculated at the pixel scale to expound the different characteristics of precipitation (Fig. S1(b)–(d)). The MAP, FPD, PCI were used to estimate the impact of the precipitation amount, precipitation frequency and precipitation distribution on precipitation-vegetation relationship in this study, respectively. FPD was defined as the ratio between the number of precipitation days and the total number of days in a year^77^, which can be directly estimated based on CRU TS4.01 rainday-counts (WET) dataset. PCI was known as a statistical derived index to quantify the relative distribution of the precipitation pattern^78^, and was defined as

2 $$PCI=100\frac{{\sum }_{i=1}^{12}\,{p}_{i}^{2}}{{({\sum }_{i=1}^{12}{p}_{i})}^{2}}$$

where *p*_*i*_ stands for the amount of precipitation for the *i*th month.

### Land cover/use and ecoregions

Land cover classification data were taken from the UMd (the University of Maryland) land cover map^79^. Built-up areas were excluded from the analysis. For detailed analysis, a map of the terrestrial ecoregions was used to identify areas with a distinct assemblage of natural communities and species^80^. To easily document potential spatial variations resulted from different biogeographical conditions, the broad land cover classes were split into ecological zones and each zone was located exclusively in one terrestrial ecoregion. In other words, the location of ecological zones was obtained from the intersection of one land use/cover class and one terrestrial ecoregion. Ecological zone across China was aggregated to a 0.5° grid to match the resolution of the climate and NDVI dataset (Fig. S2).

### Soil and topography data

Soil bulk density (SBD) and profile available water capacity (PAWC) were generated by the SoilData System, which was developed by the Global Soil Task Group of the International Geosphere-Biosphere Programme Data and Information System (IGBP-DIS)^81^. All soil datasets were at 5 arc-minutes, and were resampled to 0.5° spatial resolution to be consistent with other datasets (Fig. S3(a),(b)).

Topography database used in this study was the HYDRO1k product derived from GTOPO30 with a native 0.5° spatial resolution^82^ (Fig. S3(c),(d)). Elevation and compound topographic index (CTI) were selected to represent topography-based factors. CTI was a function of the upstream contributing area and the slope, and was correlated with zones of surface saturation and soil water content^83^. High CTI values denoted the positions with large catchments and gentle slopes^35^.

### Statistical analyses

Climate drivers, including temperature, solar radiation and precipitation interact to impose complex impacts and varying constraints on vegetation growth^19^. The simple correlation analysis may introduce bias during the analysis of the relationship between vegetation dynamics and precipitation variability. Hence, in order to remove the impacts of temperature and radiation, the second-order partial correlation coefficient between the precipitation and NDVI anomalies was calculated, with the other two climate factors (i.e. temperature and radiation anomalies) acting as control variables. The formula was given by:

3 $${r}_{12,34}=\frac{{r}_{12,3}-{r}_{14,3}\times {r}_{24,3}}{\sqrt{1-{{r}_{14,3}}^{2}}\times \sqrt{1-{{r}_{24,3}}^{2}}}$$

where *r*_*1*2,34_ refers to the partial correlation coefficient of variables 1 and 2 after fixing variables 3 and *4*; and *r*_12*,3*_ refers to the first-order partial correlation coefficient of variables *1* and *2* which uses *3* as control variables.

The first-order partial correlation coefficient was computed as follows:

4 $${r}_{12,3}=\frac{{r}_{12}-{r}_{13}\times {r}_{23}}{\sqrt{1-{{r}_{13}}^{2}}\times \sqrt{1-{{r}_{23}}^{2}}}$$

where *r*_*12*_, *r*_13_, *r*_23_ is the Pearson’s correlation coefficients between variables 1 and 2, 1 and 3, and 2 and 3, respectively.

Therefore, the spatiotemporal analysis of relationship between precipitation and vegetation variability was based on multi-temporal partial correlation analyses. At the pixel scale, correlations between the time-series of precipitation anomalies and the time-series of NDVI anomalies were expressed in terms of second-order partial correlation coefficient, controlling for temperature and radiation variables. The significant tests of the partial correlation coefficients were based on t-tests at a significance level of 95%. With strong atmospheric variability, the precipitation-vegetation relationship was better inferred from the lagged, rather than the simultaneous^84^. To assess possible delay of vegetation response to precipitation and possible impacts of different precipitation cumulation periods, the lagged cross-correlation analyses were employed. For each month during the growing season (April-October), partial correlation coefficients between NDVI and precipitation anomalies were calculated with precipitation in four different time lags (Lag 0–3 months) and six different cumulation periods (CumPer 1–6 months). As shown in Table 1, considering all combinations of time scale (lag and cumulation period) of precipitation, 168 (7×24) partial correlation analyses were carried out to determine temporal response of vegetation to precipitation anomalies:

5 $${r}_{i,j}=cor(NDVIan{o}_{i},PRan{o}_{i,j})\,4\le i\le 10,\,1\le j\le 24$$

6 $${r}_{j}=ma{x}_{4\le i\le 10}({r}_{i,j})$$

7 $${r}_{max}=ma{x}_{1\le j\le 24}({r}_{j})$$

where *cor* is the second-order partial correlation: *i* represents the *i*th month, spanning from 4th to 10th month; *j* is the combination of time scale (see Table 1); *NDVIano*_*i*_ is the ith month NDVI anomaly series; *PRano*_*i,j*_ is the *i*th month precipitation anomaly series with *j*th kind of time interval.

Hence, in this study, to remove the influence of vegetation phenology on results, monthly correlations were summarized at the growing season scale (i.e. 24 correlation analysis results). According to the resulting 24 spatial datasets of *r*_*j*_ *(1* ≤ *j* ≤ *24)*, we got the strength of relationship for a specific temporal pattern (a specific time lag and precipitation cumulation period). For each grid, the maximum partial correlation coefficient (*r*_*max*_) was obtained to evaluate the overall strength of precipitation-vegetation relationship regardless of temporal pattern.

Furthermore, to explore the seasonal difference of precipitation-vegetation relationship, pixel-based partial correlation coefficients between seasonal NDVI and the current season’s precipitation anomalies were calculated. Simultaneously, considering the lagged response of vegetation and the effects of accumulated precipitation, correlation analyses between seasonal NDVI and precipitation anomalies in the previous season, from the previous season to the current season, and in the previous two seasons were also performed.

When exploring the spatial pattern of precipitation-vegetation correlation along the precipitation (amount, frequency, and distribution) gradient, binned average analysis^43^ was carried out to minimize the uncertainty induced by other factors (such as difference in soil and topography property) that randomly influence vegetation growth.

## Supplementary information

Supplementary Information.

## Data Availability

All datasets used in this study are available online free of charge. All data and scripts are available from the corresponding author upon reasonable request.
