# Supplementary material for: Vegetation response to precipitation anomalies under different climatic and biogeographical conditions in China
Source: Sci Rep. 2020 Jan 21;10:830. doi: 10.1038/s41598-020-57910-1 (PMC6972909; doi:10.1038/s41598-020-57910-1)
Supplement: Supplementary file 1 — Supplementary Information. [file 41598_2020_57910_MOESM1_ESM.docx]

**Vegetation response to precipitation anomalies under different climatic and biogeographical conditions in China**

Zefeng Chen^a,b^, Weiguang Wang^a,b^*, Jianyu Fu^a,b^

1. *State Key Laboratory of Hydrology-Water Resources and Hydraulic Engineering, Hohai University, Nanjing 210098, China*
2. *College of Water Resources and Hydrology, Hohai University, Nanjing 210098, China*

*Corresponding author:

Dr. Weiguang Wang

State Key Laboratory of Hydrology-Water Resources and Hydraulic Engineering

Hohai University, Nanjing 210098, China

Tel +86-25-83786786

Fax: +86-25-83786786

Email: [wangweiguang2016@126.com](mailto:wangweiguang2016@126.com); wangweiguang006@126.com

# Supplementary Information

Figure S1. Spatial patterns of (a) growing season mean normalized difference vegetation index (NDVI), (b) mean annual precipitation (MAP), (c) fraction of precipitation days (FPD), and (d) precipitation concentration index (PCI) in China during the 34-year period (1982-2015).

Figure S2. Terrestrial ecoregions of China and areas of land cover/use at 0.5°×0.5° spatial resolution. Inset shows locations of meteorological stations across the country.

Figure S3. Spatial pattern of (a) soil bulk density (SBD), (b) profile available water capacity (PAWC), (c) elevation, and (d) compound topographic index (CTI) across China.

Table S1. Median value of maximum partial correlation coefficients (PCC) between precipitation and NDVI anomalies, for different ecological zones and precipitation conditions.

Table S2. Median value of partial correlation coefficients (PCC) between seasonal NDVI and precipitation anomalies, for different precipitation conditions and ecological zones.


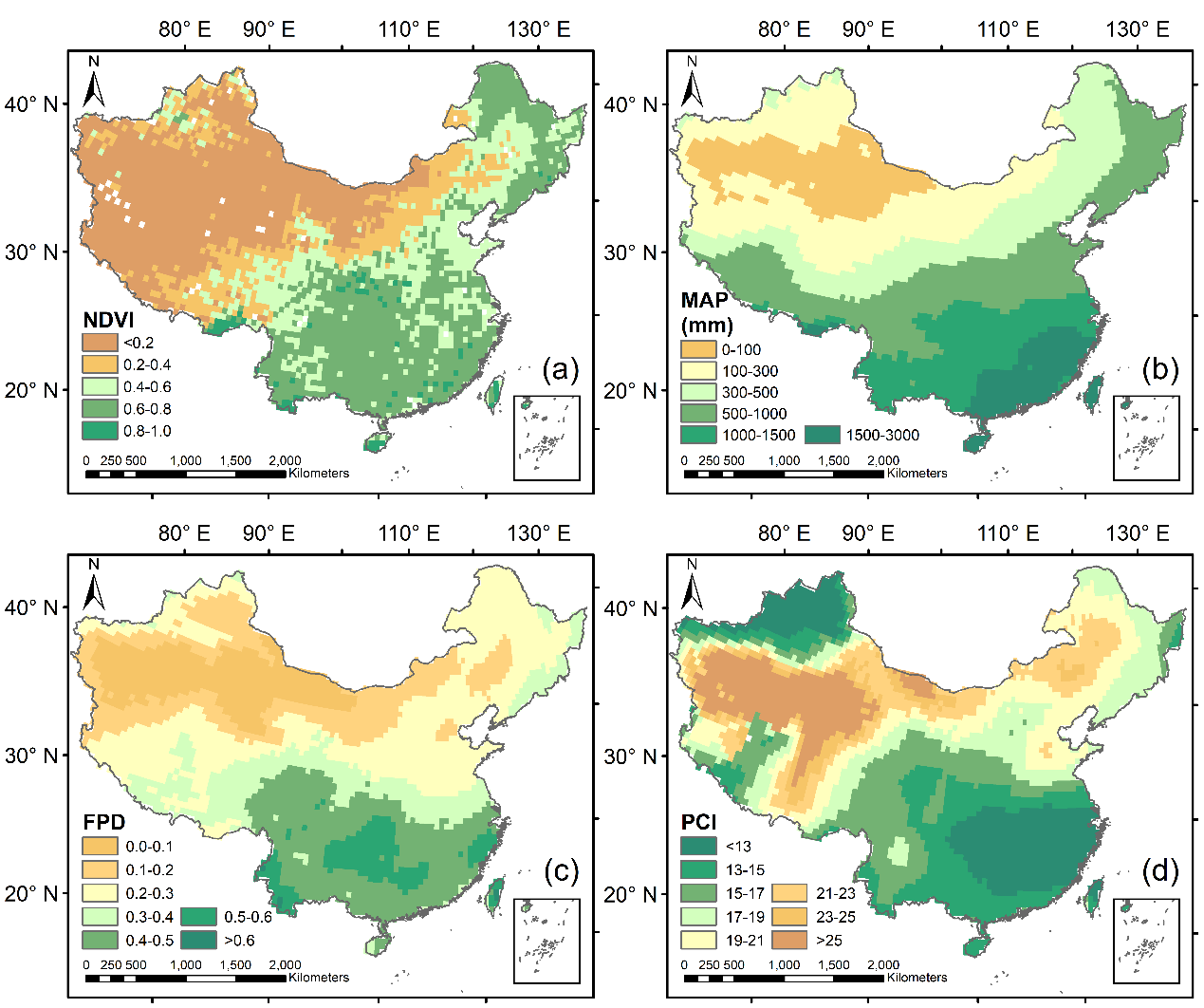


Figure S1. Spatial patterns of (a) growing season mean normalized difference vegetation index (NDVI), (b) mean annual precipitation (MAP), (c) fraction of precipitation days (FPD), and (d) precipitation concentration index (PCI) in China during the 34-year period (1982-2015).


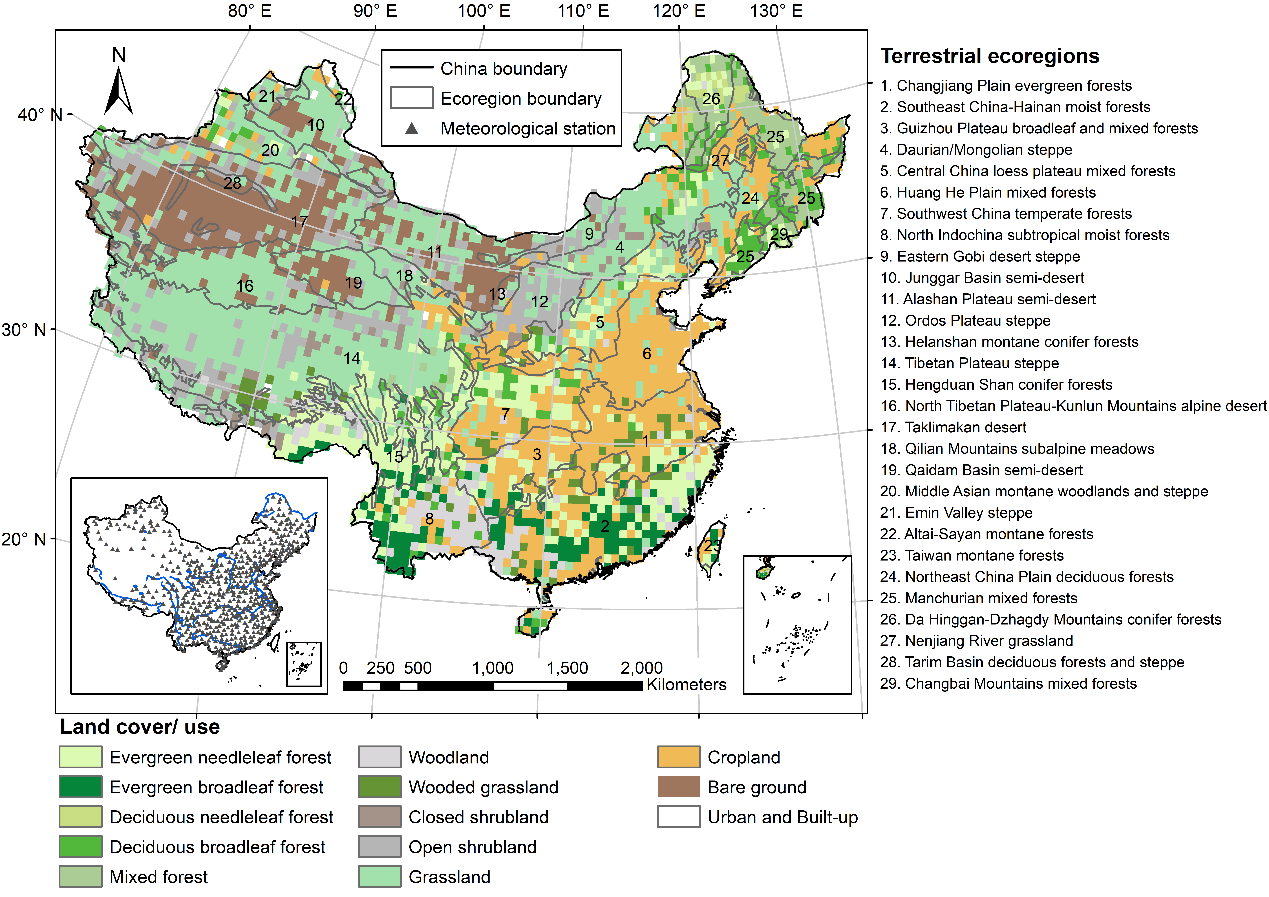


Figure S2. Terrestrial ecoregions of China and areas of land cover/use at 0.5°×0.5° spatial resolution. Inset shows locations of meteorological stations across the country.


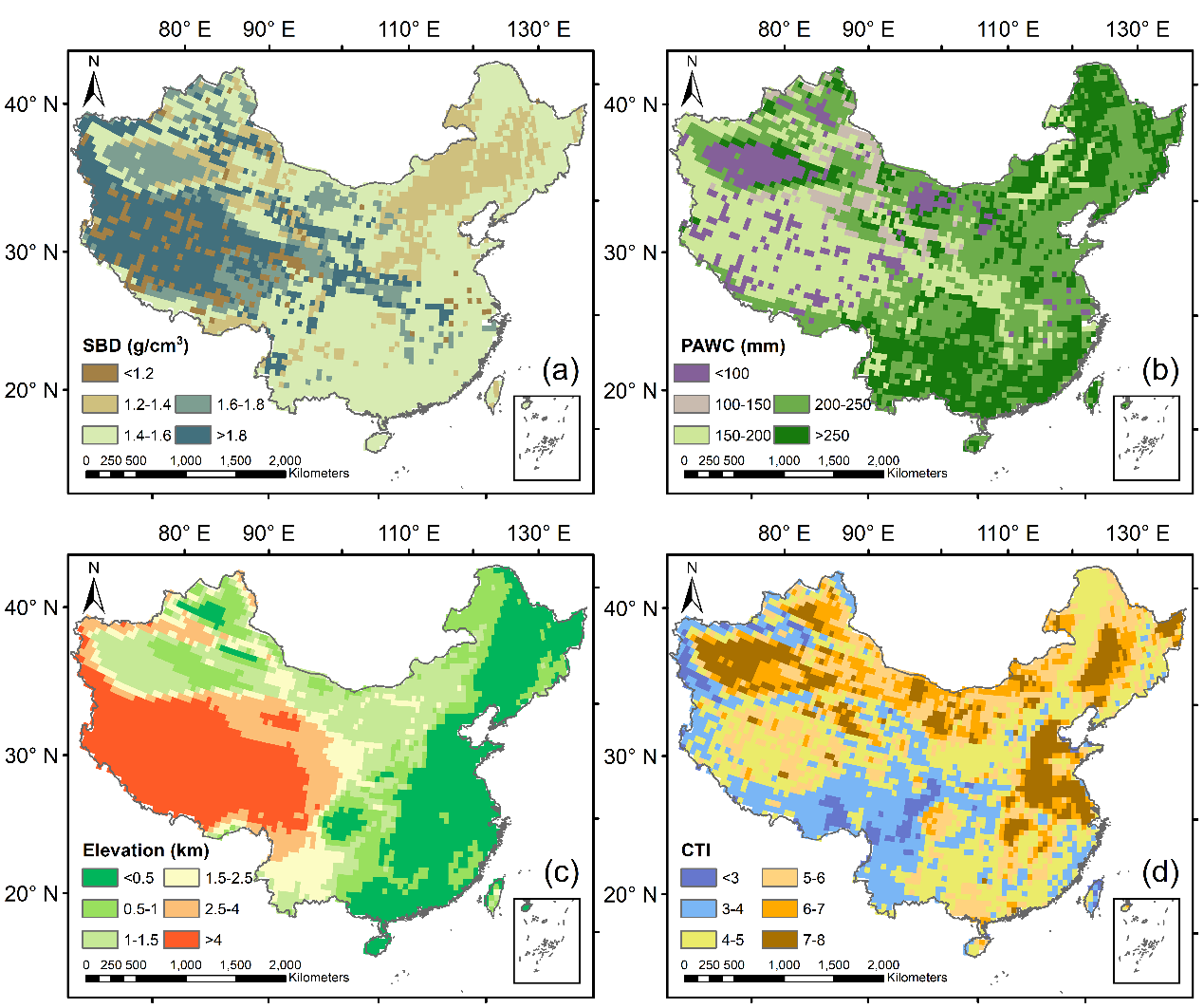


Figure S3. Spatial pattern of (a) soil bulk density (SBD), (b) profile available water capacity (PAWC), (c) elevation, and (d) compound topographic index (CTI) across China.

Table S1. Median value of maximum partial correlation coefficients (PCC) between precipitation and NDVI anomalies, for different ecological zones and precipitation conditions.

| Ecological zones | Total | Precipitation conditions | |
| --- | --- | --- | --- |
|  |  | MAP:150-500mm | MAP: 150-500mm  FPD: 0.075-0.275  PCI: 19-23 |
| Deciduous broadleaf forest (MS) | 0.45 | 0.45 *(23)* | 0.46 *(19)* |
| Mixed forest (MS) | 0.39 | 0.39 *(20)* | 0.42 *(10)* |
| Open shrubland (EGDS) | 0.67 | 0.67 *(19)* | 0.67 *(18)* |
| Open shrubland (APSD) | 0.51 | 0.55 *(19)* | 0.66 *(12)* |
| Open shrubland (OPS) | 0.58 | 0.58 *(50)* | 0.60 *(13)* |
| Open shrubland (TPS) | 0.44 | 0.45 *(39)* | 0.46 *(15)* |
| Grassland (MS) | 0.55 | 0.55 *(135)* | 0.57 *(97)* |
| Grassland (EGDS) | 0.69 | 0.69 *(16)* | 0.69 *(16)* |
| Grassland (TPS) | 0.41 | 0.44 *(227)* | 0.41 *(33)* |
| Grassland (KMAD) | 0.43 | 0.43 *(56)* | 0.43 *(11)* |
| Cropland (MS) | 0.49 | 0.49 *(44)* | 0.52 *(31)* |
| Bare ground (APSD) | 0.54 | 0.55 *(18)* | 0.57 *(12)* |

The number of grid points used in the computation is in italics.

Table S2. Median value of partial correlation coefficients (PCC) between seasonal NDVI and precipitation anomalies, for different precipitation conditions and ecological zones.

|  |  | Spring | | | | Summer | | | | Autumn | | | |
| --- | --- | --- | --- | --- | --- | --- | --- | --- | --- | --- | --- | --- | --- |
|  |  | Ra_0_ | Ra_-1_ | Ra_(-1,0)_ | Ra_(-2,-1)_ | Ra_0_ | Ra_-1_ | Ra_(-1,0)_ | Ra_(-2,-1)_ | Ra_0_ | Ra_-1_ | Ra_(-1,0)_ | Ra_(-2,-1)_ |
| All study areas | | 0.03 | 0.04 | 0.04 | 0.06 | 0.07 | 0.10 | 0.07 | 0.11 | 0.06 | 0.10 | 0.12 | 0.11 |
| Specific areas  (MAP:150-500mm) | | 0.04 | 0.06 | 0.08 | 0.14 | 0.10 | 0.13 | 0.12 | 0.16 | 0.07 | 0.14 | 0.18 | 0.17 |
| Specific areas  (MAP:150-500mm FPD:0.075-0.275 PCI:19-23) | | 0.09 | 0.07 | 0.15 | 0.15 | 0.16 | 0.13 | 0.18 | 0.17 | 0.10 | 0.18 | 0.22 | 0.20 |
| Ecological zones | Deciduous broadleaf forest (MS) | -0.04 | -0.07 | 0.03 | 0.08 | 0.22 | 0.03 | 0.18 | 0.02 | 0.00 | 0.25 | 0.24 | 0.26 |
|  | Mixed forest (MS) | -0.12 | 0.10 | 0.02 | 0.11 | 0.05 | -0.03 | 0.02 | -0.08 | -0.06 | 0.12 | 0.09 | 0.10 |
|  | Open shrubland (EGDS) | 0.38^**^ | 0.12 | 0.53^***^ | 0.22 | 0.45^***^ | 0.22 | 0.52^***^ | 0.31^*^ | 0.18 | 0.51^***^ | 0.53^***^ | 0.52^***^ |
|  | Open shrubland (APSD) | 0.34^**^ | -0.04 | 0.38^**^ | 0.22 | 0.19 | 0.36^**^ | 0.26 | 0.42^**^ | 0.17 | 0.30^*^ | 0.34^**^ | 0.34^**^ |
|  | Open shrubland (OPS) | 0.25 | 0.03 | 0.32^*^ | 0.23 | 0.28 | 0.31^*^ | 0.31^*^ | 0.41^**^ | 0.19 | 0.18 | 0.32^*^ | 0.17 |
|  | Open shrubland (TPS) | -0.01 | 0.03 | -0.05 | 0.14 | -0.02 | 0.29^*^ | 0.10 | 0.21 | -0.15 | 0.20 | 0.19 | 0.27 |
|  | Grassland (MS) | 0.16 | 0.06 | 0.19 | 0.16 | 0.28 | 0.11 | 0.29^*^ | 0.14 | 0.06 | 0.29^*^ | 0.33^*^ | 0.32^*^ |
|  | Grassland (EGDS) | 0.30^*^ | 0.26 | 0.51^***^ | 0.15 | 0.41^**^ | 0.14 | 0.51^***^ | 0.25 | 0.13 | 0.54^***^ | 0.51^***^ | 0.54^***^ |
|  | Grassland (TPS) | -0.20 | 0.05 | -0.15 | 0.03 | -0.10 | 0.00 | -0.14 | 0.00 | -0.19 | 0.12 | 0.13 | 0.06 |
|  | Grassland (KMAD) | -0.25 | 0.07 | -0.10 | 0.06 | 0.10 | -0.10 | 0.05 | -0.09 | -0.19 | 0.02 | -0.05 | 0.01 |
|  | Cropland (MS) | 0.08 | 0.09 | 0.17 | 0.22 | 0.24 | 0.04 | 0.22 | 0.04 | 0.17 | 0.25 | 0.29^*^ | 0.24 |
|  | Bare ground (APSD) | 0.24 | -0.03 | 0.26 | -0.02 | -0.07 | 0.14 | -0.05 | 0.26 | 0.13 | 0.35^**^ | 0.31^*^ | 0.33^*^ |

Note: “*”, “**”, and “***” indicates 10%, 5% and 1% significant levels of the correlation coefficient, respectively.
